# Genetic approaches to identify pathological limitations in aortic smooth muscle contraction

**DOI:** 10.1371/journal.pone.0193769

**Published:** 2018-03-01

**Authors:** Jian Huang, Ning Gao, Shanzhi Wang, Dianna M. Milewicz, Kristine E. Kamm, James T. Stull

**Affiliations:** 1 Department of Physiology, University of Texas Southwestern Medical Center, Dallas, TX United States of America; 2 Department of Internal Medicine, University of Texas Health Science Center at Houston, Houston, TX United States of America; Cinvestav-IPN, MEXICO

## Abstract

Aortic smooth muscle contains limiting amounts of myosin light chain kinase (MLCK) for myosin regulatory light chain (RLC) phosphorylation and contraction that predisposes to thoracic aortic disease in humans containing heterozygous loss-of-function mutations in *MYLK*. We tested the hypothesis that thoracic aortic smooth muscle contraction may also be susceptible to variations in the smooth muscle-specific isoform of the motor protein myosin where inactivation of one *Myh11* allele or the presence of one *Myh11* missense variant associated with an increased risk of human aortic disease may result in a reduced force development response. Additionally, other kinds of smooth muscles may be less sensitive to the effects of mutations in one smooth muscle myosin allele, similar to results obtained with *Mylk*. Force development responses were reduced in aortic tissue from a conditional knockout of smooth muscle myosin heavy chain in adult mice (*Myh11*^+/- ^or *Myh11*^-/-^) with a greater reduction with homozygous vs heterozygous tissues. Similar reductions in force responses were obtained with tissues containing either a heterozygous or homozygous knockin mutation in smooth muscle myosin heavy chain (*Myh11*^+/R247C^ or *Myh11*^R247C/R247C^ mutations that cause human aortic disease) with no significant changes in RLC phosphorylation. Agonist-dependent force responses were not reduced significantly in urinary bladder, ileal, or tracheal tissues from *Myh11*^+/-^ mice while only ileal tissue showed a reduced force response in *Myh11*^R247C/R247C^ mice. Thus, heterozygous mutations in *Myh11* associated with reduced myosin function result in compromised contractile function primarily in aortic smooth muscle.

## Introduction

Mutations in genes encoding proteins of the contractile/signaling modules of smooth muscle cells, including *ACTA2* (smooth muscle α-actin), *MYH11* (smooth muscle myosin heavy chain), *MYLK* (myosin light chain kinase), and *PRKG1* (PKG type-1) [1–4], predispose to thoracic aortic disease with aneurysms and acute ascending aortic dissections in humans [5]. The identification of contractile and signaling proteins affecting force development involvement in aortic disease may indicate a lifelong role for cellular mechanotransduction to maintain aortic structure and function [6–10].

The thoracic aorta segment is exposed to the highest biomechanical forces in the circulation arising from pulsatile ejection of blood by the heart. The aortic smooth muscle cell, thus, occupies a continuously dynamic environment with imposed stresses distinct from smooth muscle cells in the gastrointestinal system, urinary bladder, airways, and other smooth muscle tissues. However, the primary smooth muscle contractile proteins α-actin, myosin (a hexamer of two each of heavy chains, essential light chain and RLC), and MLCK are expressed in all smooth muscle-dependent organs of the body [11, 12]. The physiological environment combined with loss-of-function mutations in proteins of the contractile system in aortic smooth muscle cells may contribute to the propensity of the aorta to disease [5, 13–15].

Phosphorylation of myosin RLC by MLCK is required for the cyclic interaction of myosin with actin that leads to contraction (force development) in smooth muscle [11, 15–20]. Diverse cell signaling pathways increase smooth muscle cell intracellular Ca^2+^ concentrations, [Ca^2+^]_i_. Ca^2+^ binding to calmodulin leads to MLCK activation and RLC phosphorylation to initiate smooth muscle contraction [11, 16, 21–25]. Myosin light chain phosphatase dephosphorylates RLC, and induces relaxation after a decrease in [Ca^2+^]_i_ returns MLCK to an inactive state [11, 17].

Genetic approaches have revealed essential roles for myosin and MLCK in smooth muscle contraction. Aortic tissues from homozygous *Myh11*^R247C/R247C^ mice containing a rare variant that in humans is associated with increased risk of thoracic aortic aneurysms and dissections demonstrated reduced contractile responses [14]. Contractile responses of other smooth muscle tissues were not examined. In isolated neonatal bladder preparations from homozygous mice containing a conventional knockout of *Myh11*, there was no initial force transient and the sustained force was only 11%, presumably from nonmuscle myosin in smooth muscle cells [26–29]. Death occurred within a few days after birth. The conditional homozygous knockout of MLCK in smooth muscle cells in adult mice was also lethal because of contractile failure in different smooth muscles *in vivo* with greatly diminished force responses in different isolated smooth muscle tissues [15, 18–20].

Interestingly, inactivation of only one *MYLK* allele in humans induces aortic dissections, and the partial reduction of MLCK content in smooth muscle tissues from heterozygous MLCK^SM+/-^ mice led to a reduction of RLC phosphorylation and force responses in aortic, but not bladder, airway, or ileal smooth muscle tissues [3, 15, 18, 19]. Thus, aortic tissue showed a unique sensitivity to reduced MLCK content, probably related to the limiting amount of MLCK in aortic, but not other smooth muscles.

Mutations in one smooth muscle myosin allele may predispose to thoracic aortic disease with aneurysms and acute ascending aortic dissections in humans [5]. We considered the possibility that inactivation of one *Myh11* allele or the presence of one *Myh11* missense variant associated with human aortic disease may result in the reduction of force development response in thoracic aorta. Additionally, other kinds of smooth muscles may be less sensitive to the effects of mutations in one smooth muscle myosin allele. These hypotheses were tested in genetically modified mice.

## Materials and methods

### Generation of genetically modified mice

*Myh11*^R247C/R247C^ knockin mice (denoted as *Myh11*^R/R^) containing mutated DNA within two loxP sites were previously described [14]. Mice were crossed with a transgenic SMA-Cre-ER^T2^ mouse line in which the expression of the tamoxifen-dependent Cre-ER^T2^ recombinase is under the control of a large genomic DNA segment of the mouse smooth muscle α-actin (SMA) gene [30], resulting in *Myh11*^*R247C/R247C*^, Cre+ mice. The Cre-ER^T2^-mediated recombination of LoxP flanked target DNA is strictly tamoxifen-dependent, and efficient in both vascular and visceral smooth muscle cells, including the aorta. Mice were bred and screened as described previously [31–33]. Male mice (8–10 weeks old) were injected intraperitoneally with tamoxifen or vehicle for five consecutive days each week for two weeks at a dose of 1 mg/day[33].

Experiments were performed in accordance with the National Institutes of Health and Institutional Animal Care and Use Guidelines. The Institutional Animal Care and Use Committee at the University of Texas Southwestern Medical Center approved all procedures and protocols in compliance with recommended physiological guidelines (Drummond, 2009). Animals were administered an intraperitoneal lethal dose of tribromoethanol (250 mg/kg) for tissue collection.

### Force measurements

Thoracic aortas isolated from anesthetized mice were dissected free of excessive adventitia, and endothelial cells were removed by gentle swabbing. Thoracic aortic segmental rings 5 mm long were mounted by triangular wires to an isometric force apparatus. Aortic rings were passively stretched to 1.8–2.0 g and remained quiescent for 60 min before being contracted with 65 mm KCl in Krebs-Ringer solution followed by treatment with 10 μM phenylephrine. Force responses were recorded isometrically by a Grass FT03 force transducer connected to Powerlab 8/SP data acquisition unit (AD Instruments, Colorado Springs, CO). Force measurements were normalized as grams of developed force per tissue wet weight. Tracheal rings of mice were isolated and connective tissue removed for force measurements as previously described [19]. Urinary bladder and ileal smooth muscle tissues were prepared for force measurements as previously described [25, 34]. Phenylephrine and carbachol were used as agonists for aortic tissue vs other smooth muscles respectively to elicit RhoA/ROCK signaling that would affect force development compared to KCl used to depolarize smooth muscle cells for Ca^2+^ influx.

### Protein analyses

At indicated times after specific treatments, tissues were quick frozen by clamps pre-chilled in liquid nitrogen. Frozen muscles were processed as described previously by immersion in a frozen slurry of 10% trichloroacetic acid in acetone containing 10 mM dithiothreitol for 30 min, then thawed slowly at room temperature and transferred to microcentrifuge tubes. Tissues were rinsed three times with diethylether, briefly exposed to air for dispersion of residual diethylether, and suspended in urea sample buffer containing 8 M urea, 18.5 mM Tris (pH 8.6), 20.4 mM glycine, 10 mM dithiothreitol, 4 mM EDTA, and 5% sucrose. Proteins were then solubilized in a Bullet Blender (Next Advance, Inc., Averill Park, NY) (with 2-mm zirconium oxide beads, four spins × 3-min each at setting 9) and protein measured by Bradford assay with bovine serum albumin as the standard. Bromophenol blue was added to 0.004% and the sample was stored at -80°C. Aortic samples were quick frozen at 15 secs for maximal phosphorylation response which precedes the early maximal force response [15].

Protein samples were subjected to electrophoresis (4–15% SDS-PAGE) followed by staining 0.1% Coomassie Blue R250 in 10% acetic acid, 50% methanol, and 40% H_2_O with shaking overnight. Gels were destained by rocking for 3 to 4 hours in 10% acetic acid, 50% methanol, and 40% H_2_O with solution changes, then stored in 10% acetic acid. Myosin heavy chain and actin contents were determined by quantitative densitometry using the ImageQuantTL software package (GE). Data were normalized to the amount of protein in samples from wildtype mice for each tissue.

RLC phosphorylation was measured by urea/glycerol-PAGE as previously described [15, 35]. Muscle proteins in 8 M urea sample buffer were subjected to urea/glycerol-PAGE to separate nonphosphorylated and monophosphorylated RLC. Because of the reduction in RLC amount in tissues from *Myh11*^-/-^ or *Myh11*^+/-^ mice, additional protein was loaded for electrophoresis to improve the sensitivity of the phosphorylation measurements. Following electrophoresis, proteins were transferred to PVDF membranes, and probed with rabbit polyclonal antibodies against smooth muscle RLC [36]. The urea/glycerol-PAGE system separates nonphosphorylated from the monophosphorylated RLC so direct quantitative measures of RLC phosphorylation is obtained as mol of phosphate per mol of RLC. Diphosphorylation results in additional migration of RLC in the urea-PAGE system but there is normally little diphosphorylated RCL formed in response to KCl or agonists in isolated smooth muscle tissues, including herein. Quantitative measurements were processed on a Storm PhosphorImager and analyzed by ImageQuant software.

### Statistical analyses

All data are presented as mean ± S.E.M. Statistical comparisons were performed by Student's t test for force development and RLC phosphorylation. One-way ANOVA was performed followed by Newman-Keuls post hoc test for multiple comparisons. Data analyses were performed with statistical software (GraphPad Prism Software, San Diego, CA, USA). P values less than 0.05 were considered statistically significant.

## Results

### Myosin heavy chain expression is reduced in different mouse smooth muscle tissues with Myh11 gene ablation, but not with the R247C knockin mutation

**Fig 1:**
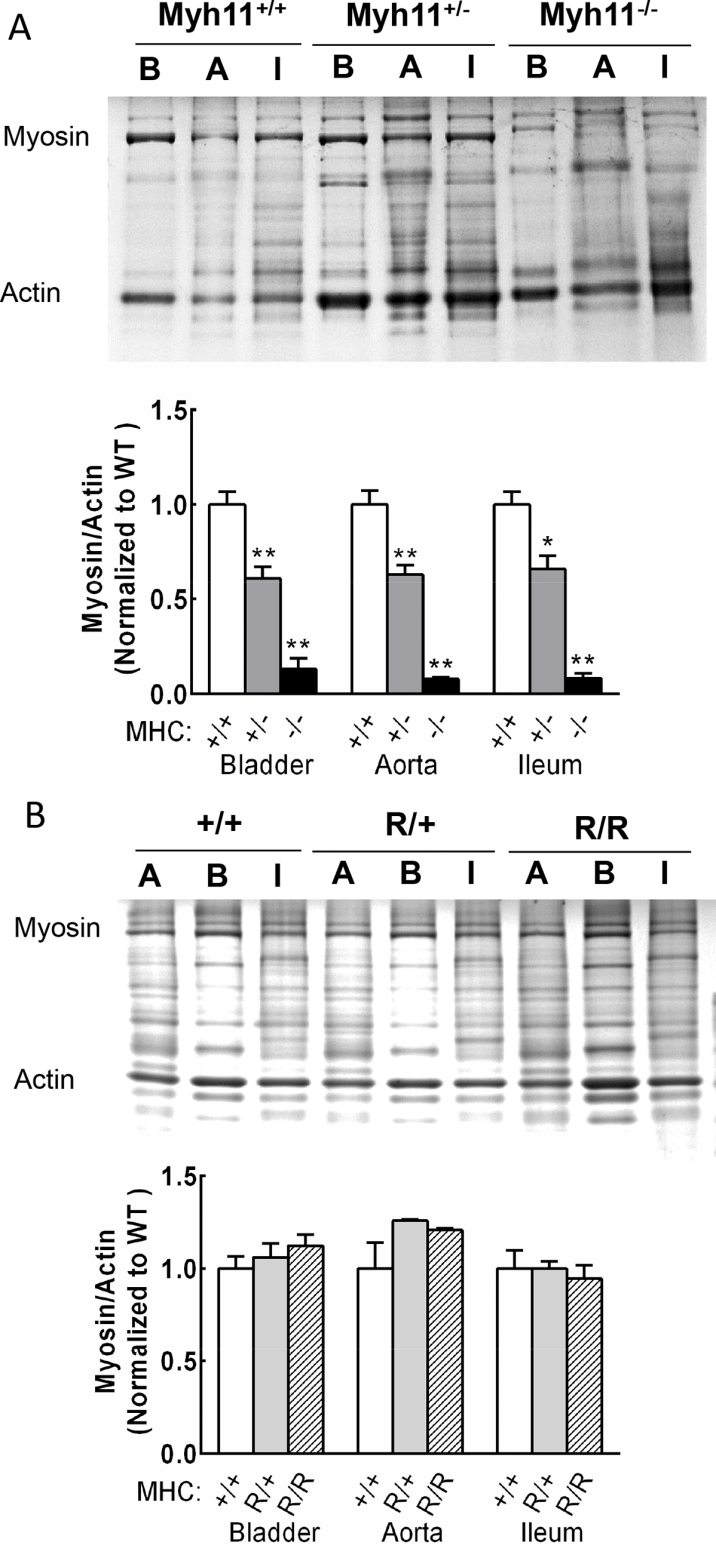
Quantification of myosin heavy chain content. A) Representative gel image of tissue homogenates from bladder, aortic and ileal tissues from *Myh11* knockout mice. Proteins separated by SDS-PAGE were stained with Coomassie blue, and myosin content was normalized to actin. Amount measured in *Myh11*^+/-^ (+/-, dark gray bars) and *Myh11*^-/-^ (-/-, black bars) tissues were normalized to the average of *Myh11*^+/+^ (+/+, open bars). B indicates bladder; A, aorta; and I, ileum; MHC, myosin heavy chain. B) Myosin heavy chain content in tissue homogenates of bladder, aorta and ileum from *Myh11* R257C mutation knockin mice. Values from *Myh11*^R247C/+^ (R/+, light gray bars) or *Myh11*^R247C/ R247C^ mice (R/R, hatched bars) were compared with values for *Myh11*^+/+^ mice (+/+, open bars); *p<0.05, **p<0.01, *N* = 3 by Student’s t test.

The conditional inactivation of both *Myh11* alleles decreased smooth muscle myosin heavy chain expression in bladder, aortic, and ileal tissues (Fig 1A). Tissues were harvested 18–20 days from these animals after starting tamoxifen injections before myosin heavy chain protein was fully extinguished because of impending death related to the essential role of myosin in maintenance of homeostasis by smooth muscle organ systems [26, 27]. With the removal of different tissues, the intestines appeared flaccid and empty while bladders were typically full, presumably due to the inability to empty [27]. These results suggest weak contractions consistent with observations described below for isolated tissues in vitro.

In contrast to the results reported for neonatal bladders containing a conventional gene ablation in smooth muscle *Myh11*^+/-^ mice [26], the bladder, aortic, and ileal tissues from the conditional knockout in *Myh11*^+/-^ mice showed reductions in myosin heavy chain content (Fig 1A). In tissues from *Myh11*^*-/-*^ mice, myosin heavy chain content in bladder, aorta and ileum decreased by 87±6%, 92±1% and 92±2%, respectively. Consistent with knockout of one allele in tissues from *Myh11*^+/-^ mice, the myosin heavy chain content in the bladder, aorta and ileum decreased by 39±6%, 37±5% and 34±7%, respectively.

As expected, myosin heavy chain content did not change in these different smooth muscle tissues from either *Myh11*^R247C/+^ or *Myh11*^R247C/R247C^ mice (Fig 1B).

### Aortic force development is reduced with Myh11 gene ablation

**Fig 2:**
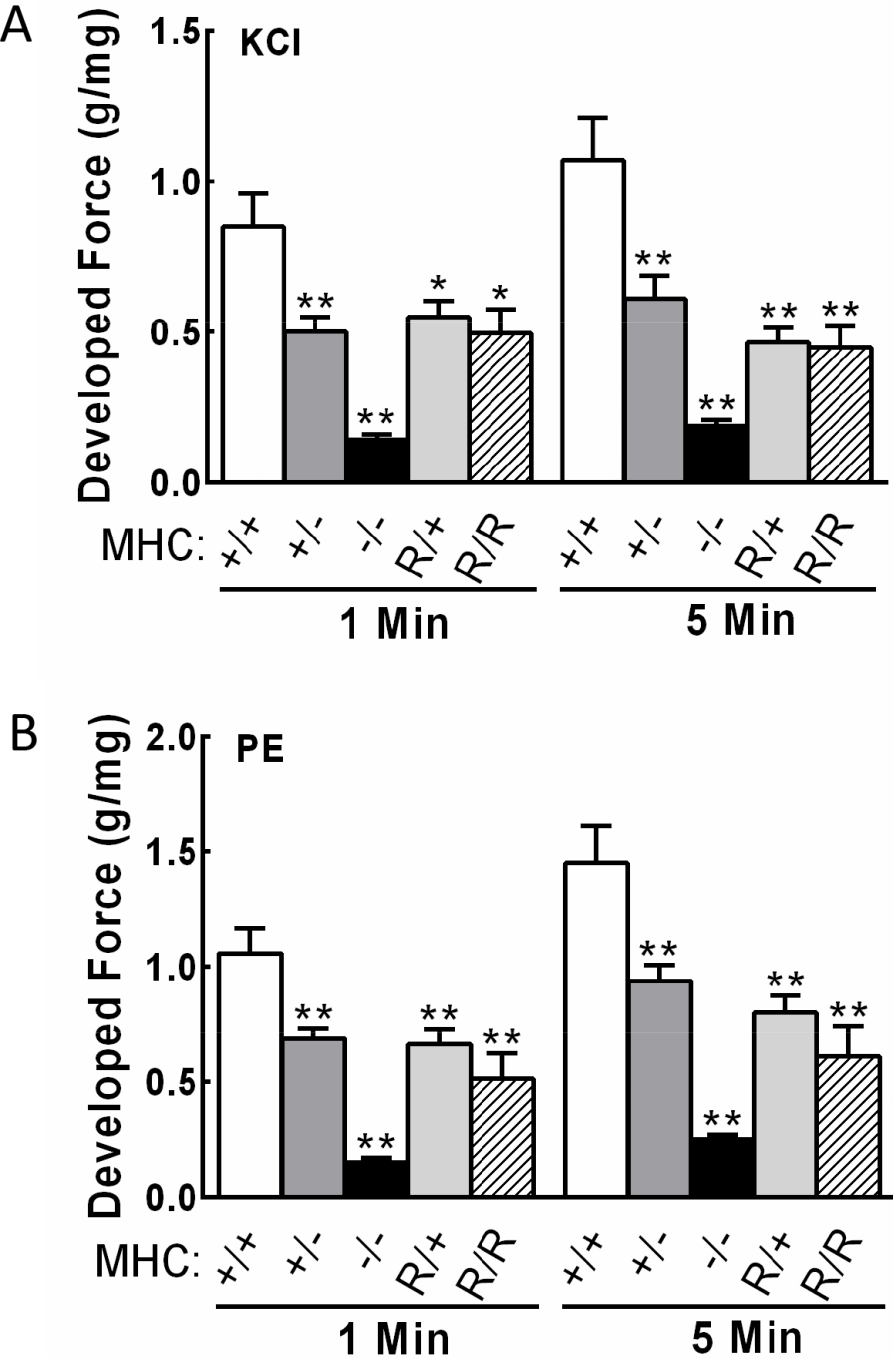
Developed force responses in aortic rings from smooth muscle myosin heavy chain mutant mice. A) KCl (65 mM) stimulated initial force responses at 1 min and sustained force responses at 5 min. B) Phenylephrine (PE)(10 μM) stimulated initial force responses at 1 min and sustained force responses at 5 minutes. Sample groups are follows: *Myh11*^+/+^ (+/+, open bars), *Myh11*^+/-^(+/-, dark gray bars), *Myh11*^-/-^ (-/-, black bars), *Myh11*^R247C/+^ (R/+, light gray bars) and *Myh11*^R247C/R247C^ (R/R, hatched bars). Significance was determined by comparisons to *Myh11*^+/+^ average at 1 min or 5 min. *p<0.05, **p<0.01, *N* ≥ 8 by One-way ANOVA followed by Newman-Kuels post hoc test for multiple comparisons.

The initial one-minute force development or sustained five-minute force responses to KCl or phenylephrine treatments were reduced to 15% in aortas from *Myh11*^-/-^ mice (Fig 2A and 2B), consistent with the marked reduction in smooth muscle myosin heavy chain content. We also found that force responses were reduced 42% in aortic tissues from *Myh11*^+/-^ mice (Fig 2A and 2B), consistent with the partial reduction in myosin heavy chain content (Fig 1A and 1B).

**Fig 3:**
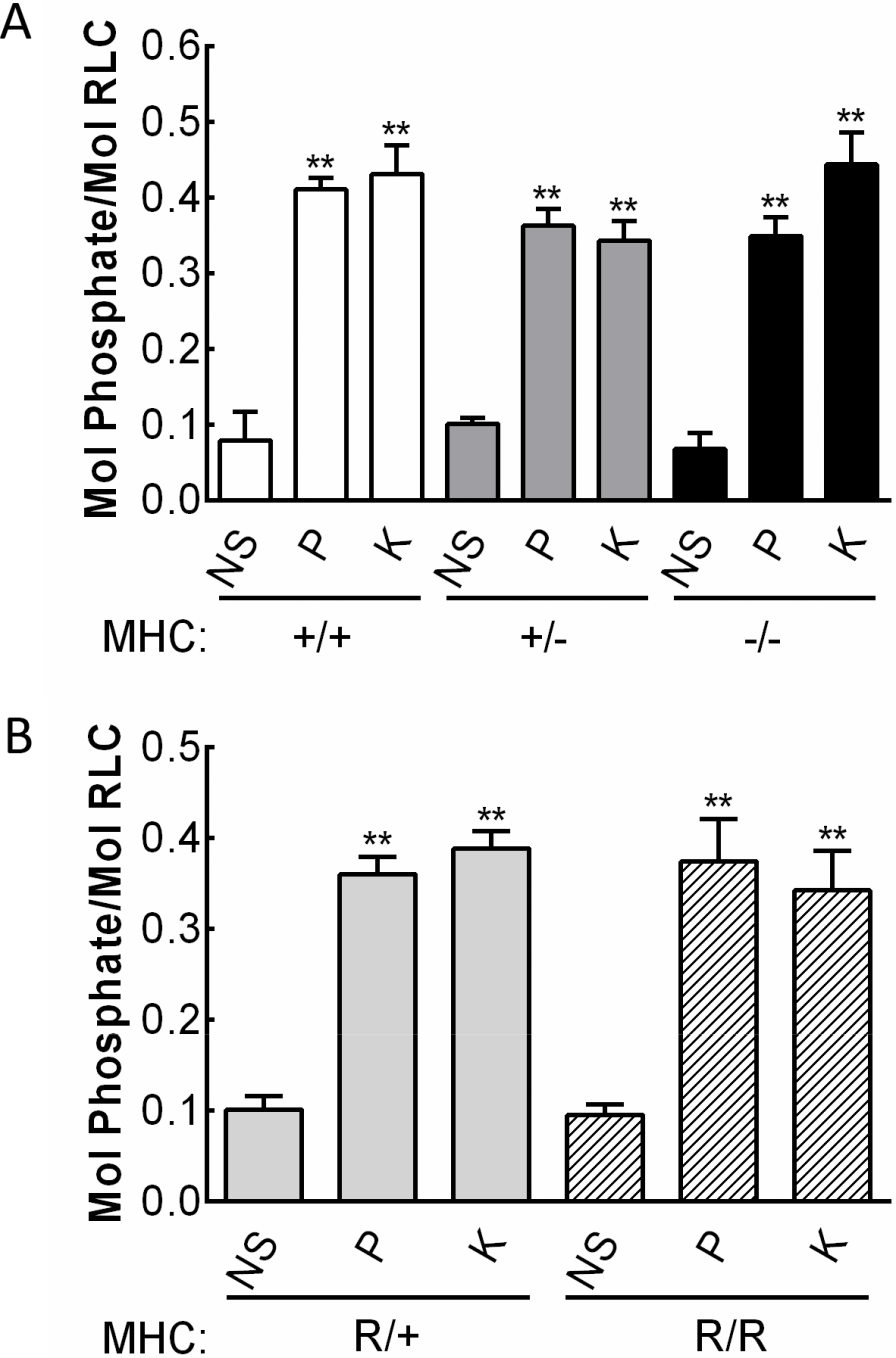
RLC phosphorylation in aortic rings from smooth muscle myosin heavy chain mutant mice. Extent of RLC phosphorylation in response to 10 μM phenylephrine (P) or 65 mM KCl (K) for 15 secs were compared to nonstimulated (NS) tissue values in A) *Myh11* knockout mice, and B) *Myh11* R247C mutant knockin mice. Experimental groups are as follows: *Myh11*^+/+^ (+/+, open bars), *Myh11*^+/-^(+/-, dark gray bars) and *Myh11*^-/-^ (-/-, black bars), *Myh11*^R247C/+^ (R/+, light gray bars) and *Myh11*^R247C/R247C^ (R/R, hatched bars). **p <0.01 compared with RLC phosphorylation at rest in nonstimulated tissues from *Myh11*^+/+^ mice (*N* = 3–7); *N* = 11–16 in all other groups by Student’s t test.

The extents of RLC phosphorylation obtained with KCl or phenylephrine treatments in aortas from *Myh11*^-/-^ or *Myh11*^+/-^ were not changed compared to tissues from wildtype animals (Fig 3A). Thus, the reduced force responses appear to be proportional to the reduced myosin content and its associated RLC subunit with a greater reduction in aortic tissue from *Myh11*^-/-^ mice compared to *Myh11*^+/-^ mice. The western blot quantification of RLC phosphorylation does not distinguish between smooth vs nonmuscle RLC. However, the homozygous knockout of the smooth muscle myosin heavy chain resulted in 8–13% of myosin remaining in the smooth muscle tissues. Thus, the amount of nonmuscle RLC is substantially less than smooth muscle RLC. Although there were no changes in the extent of RLC phosphorylation, the total amount of phosphorylated RLC would be reduced relative to the reduction in myosin heavy chain in *Myh11*^+/-^ and *Myh11*^-/-^ mice. Signaling mechanisms leading to MLCK activation and RLC phosphorylation appear not to be affected with the myosin heavy chain knockout.

### Aortic force development is reduced with the Myh11 R247C mutation

Aortic tissues homozygous for the myosin heavy chain knockin mutation R247C showed reduced force responses to KCl and phenylephrine, consistent with our previous report [14]. However, force responses in aortic rings from heterozygous *Myh11*^R247C/+^ mice were also similarly reduced (Fig 2A and 2B). These findings are particularly relevant to understanding compromised force development since this heterozygous *MYH11* R247C rare variant has been identified in patients with thoracic aortic disease.

There was also no attenuation in RLC phosphorylation in aortic rings from *Myh11*^R247C/+^ or *Myh11*^R247C/R247C^ mice (Fig 3B). Therefore, the defective contractile responses did not result from changes to signaling modules acting on RLC phosphorylation, but appear to be intrinsic to the properties of the mutated myosin.

### Force responses with Myh11 gene ablation or myosin containing the R247C mutation in different smooth muscles

**Fig 4:**
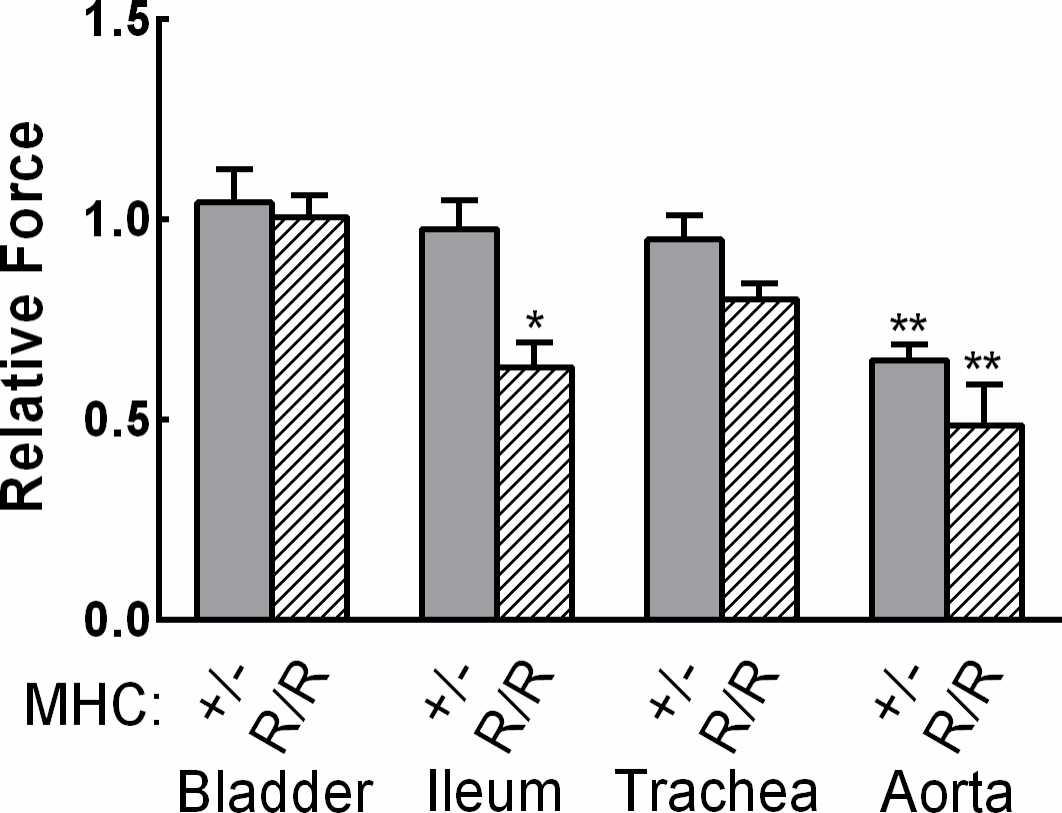
Relative force responses in different smooth muscles from myosin heavy chain mutant mice. Force responses in bladder, ileum, and trachea were calculated as a ratio of force developed in tissues from *Myh11*^+/-^ or *Myh11*^R247C/R247C^ mice to the respective tissues from *Myh11*^+/+^ mice with all tissues treated with 10 μM carbachol for 1 min. Significance of differences were determined by Student’s t-test; ** p<0.01, *N* = 5–7. Tissues were from *Myh11*^+/-^ (+/-, dark gray bars) or *Myh11*^R247C/R247C^ (R/R, hatched bars) mice. Aortic force was obtained similarly in different tissues with 10 μM phenylephrine.

Considering the attenuation of force responses in aortic but not other kinds of smooth muscles from MLCK^SM+/-^ mice [15, 18, 19], we determined force responses in bladder, ileum, and trachea from *Myh11*^+/-^ and *Myh11*^R247C/R247C^ mice (Fig 4). Similar forces were obtained in response to agonist with no significant attenuation compared to wildtype mice for bladder, ileal, and tracheal tissues from *Myh11*^+/-^ mice while there was a significant force reduction response in aorta from *Myh11*^+/-^ mice. There was also no reduction in force responses in bladder or tracheal tissues from *Myh11* ^R247C/R247C^ mice compared to responses from wildtype tissues. However, there was a reduction in the force response for ileal tissues. Collectively, the aortic smooth muscle cell appears particularly sensitive to compromised force responses with mutations in *Myh11* genes, similar to observations with MLCK.

## Discussion

Through genetic approaches we investigated the properties of aortic smooth muscle contraction associated with changes in smooth muscle myosin. The conditional homozygous knockout of smooth muscle myosin heavy chain in adult mice led to a profound reduction in myosin content as well as force responses in aortic, bladder, and ileal smooth muscle similar to results obtained with neonatal bladders from a conventional myosin heavy chain knockout [26, 27]. The conditional heterozygous myosin heavy chain knockout partially reduced myosin heavy chain content with a proportional decrease in the force response without a change in the extent of RLC phosphorylation. Thus, there appear to be no changes in Ca^2+^ signaling pathways to RLC phosphorylation, and the force reduction may be explained by the reduction in the amount of myosin in aortic smooth muscle cells. These results would be consistent with heterozygous loss-of-function myosin mutations potentially leading to compromised force development in humans that may predispose to aortic disease [5, 7, 14].

Force production may also be affected by mutations in myosin heavy chain that alter its biochemical properties. The myosin mutation R247C located in the head region of the myosin heavy chain decreased maximal actin-activated myosin ATPase activity with a reduction in release of ATP hydrolysis products ADP and phosphate when RLC was phosphorylated [14]. The mutant myosin had no activity in the absence of RLC phosphorylation as expected for regulation by phosphorylation. These biochemical properties are consistent with the reduction in aortic force for tissues from *Myh11*^R247C/R247C^ mice presented herein and previously published [14]. Importantly, force development was also reduced for aortas from *Myh11*^R247C/+^ mice. Because the extent of RLC phosphorylation was similar in tissues from wildtype and mutant mice, it probably followed a random phosphorylation process without a selective phosphorylation of RLC on the mutant or wildtype heavy chain [36]. Also, the mutation was in the head region and similar amounts of myosin heavy chain were expressed in tissues from wildtype and mutant mice so thick filaments probably form from the random polymerization of mutant and wildtype myosin heavy chains. Thus, the altered biochemical properties of the mutated myosin appear to have a dominant negative effect on wildtype myosin in smooth muscle cells. These results predict heterozygous dysfunctional myosin mutations in humans may result in reduced force production that predisposes to thoracic aortic disease [5].

Different cellular mechanisms may be associated with aortic force reduction responses. Reduction of MLCK content by half did not significantly reduce forces in bladder, tracheal, or ileal smooth muscles [15, 18]. These results were thought to be related to differences in the amount of free Ca^2+^/calmodulin in smooth muscle cells. A genetically encoded biosensor MLCK was not saturated with Ca^2+^/calmodulin after maximal increases in [Ca^2+^]_i_ in bladder tissues [23, 24, 37] and the reduction of MLCK to half the normal amount did not affect RLC phosphorylation [15]. The normal MLCK content was less in aortic tissue, and RLC phosphorylation and force responses were reduced when MLCK was reduced 50%, suggesting in aortic smooth muscle cells, MLCK, not Ca^2+^/calmodulin, was limiting. Thus, the aortic smooth muscle cell appears particularly sensitive to the partial loss of MLCK.

Other smooth muscle tissues containing half of the normal amount of myosin or a mutated myosin R247C had no or little reduction in force development compared to responses from aorta. Thus, the aortic smooth muscle cell also appears particularly sensitive to the disrupting effects of mutations in myosin. Force development is not only dependent on smooth muscle myosin and RLC phosphorylation, but also requires the polymerization of submembranous cytoskeletal actin filaments to strengthen membrane adhesion complexes involved in transmitting force generated in actin-myosin filaments to extracellular structures [29, 38]. The integrin-containing membrane adhesome complexes on the cell surface involve many dynamic processes that couple the contractile actin-myosin system to the extracellular matrix for force transmission. Stronger adhesome complexes combined with the high elastin and collagen content as well as tissue-specific stroma structures for aorta vs other smooth muscles [39] may produce less compliant structures for force transmission by the contractile actin-myosin system where compromised myosin function is more readily revealed [5, 7]. Additionally, contractions that lead to shortening of smooth muscle cells reduce blood flow in small arteries as well as emptying the hollow organs (bladder and intestines), but do not play this role in the aorta. Rather, aortic smooth muscle cell contraction potentially functions as a mechanosensor [5, 40]. Mutations in proteins affecting the actin-myosin system of aortic smooth muscle cells independently converge to disrupt the ability of the smooth muscle cell to generate force through the elastin-contractile units in response to pulsatile blood flow. Thus, the vulnerability of the thoracic aorta to aneurysms and dissections may result from the compromised ability of the smooth muscle cells to sense their mechanical environment, and maintain and regulate the mechanical state of the extracellular matrix [5, 7].

Mutations that reduce aortic force production in mice do not produce aortic aneurysms or dissections similar to human disease for up to 10 months of age [3, 14, 15]. *Myh11*^*R247C/R247C*^ mice exhibited normal growth, reproduction, and aortic histology but decreased aortic contraction. In response to vascular injury, *Myh11*^*R247C/R247C*^ mice showed significantly increased neointimal formation due to increased SMC proliferation when compared with the wild-type mice. Although additional studies have not been performed on *Myh11*^*+/-*^ or *Myh11*^*R247C/+*^ mice we note that they live for at least six months and appear normal. In the absence of other disease modifiers, these mutations may be tolerated because the smooth muscle cells can compensate physiologically *in vivo* for the force output necessary to maintain aorta structure and function. In terms of induction of aortic disease in mice, additional studies are needed to identify additional genetic or environmental modifiers.

## Conclusion

In summary, studies with genetically modified mice show that modifications of MLCK and myosin heavy chain independently converge to disrupt the ability of the aortic smooth muscle cell to generate force. Heterozygous mutations in *Myh11* associated with reduced myosin function without changing the extent of RLC phosphorylation result in compromised contractile function in aortic smooth muscle, similar to reported results for decreased MLCK function which acts to reduce myosin function by reducing the extent of RLC phosphorylation. The aorta is particularly sensitive to compromised contractile function compared to other kinds of smooth muscles which may disrupt mechanosensing mechanisms and predispose to thoracic aorta disease.

## References

[1] Guo DC, Pannu H, Tran-Fadulu V, Papke CL, Yu RK, Avidan N, et al Mutations in smooth muscle alpha-actin (ACTA2) lead to thoracic aortic aneurysms and dissections. Nat Genet. 2007;39(12):1488–93. Epub 2007/11/13. doi: 10.1038/ng.2007.6 . 17994018 10.1038/ng.2007.6

[2] Zhu L, Vranckx R, Van Kien PK, Lalande A, Boisset N, Mathieu F, et al Mutations in myosin heavy chain 11 cause a syndrome associating thoracic aortic aneurysm/aortic dissection and patent ductus arteriosus. Nat Genet. 2006;38(3):343–9. http://www.nature.com/ng/journal/v38/n3/suppinfo/ng1721_S1.html. doi: 10.1038/ng1721 16444274 10.1038/ng1721

[3] Wang L, Guo DC, Cao J, Gong L, Kamm KE, Regalado E, et al Mutations in myosin light chain kinase cause familial aortic dissections. Am J Hum Genet. 2010;87(5):701–7. Epub 2010/11/09. doi: 10.1016/j.ajhg.2010.10.006 ; PubMed Central PMCID: PMC2978973. 21055718 10.1016/j.ajhg.2010.10.006 PMC2978973

[4] Guo DC, Regalado E, Casteel DE, Santos-Cortez RL, Gong L, Kim JJ, et al Recurrent gain-of-function mutation in PRKG1 causes thoracic aortic aneurysms and acute aortic dissections. Am J Hum Genet. 2013;93(2):398–404. Epub 2013/08/06. doi: 10.1016/j.ajhg.2013.06.019 ; PubMed Central PMCID: PMCPMC3738837. 23910461 10.1016/j.ajhg.2013.06.019 PMC3738837

[5] Milewicz DM, Trybus KM, Guo DC, Sweeney HL, Regalado E, Kamm K, et al Altered Smooth Muscle Cell Force Generation as a Driver of Thoracic Aortic Aneurysms and Dissections. Arterioscler Thromb Vasc Biol. 2017;37(1):26–34. Epub 2016/11/24. doi: 10.1161/ATVBAHA.116.303229 ; PubMed Central PMCID: PMCPMC5222685. 27879251 10.1161/ATVBAHA.116.303229 PMC5222685

[6] Milewicz DM, Guo DC, Tran-Fadulu V, Lafont AL, Papke CL, Inamoto S, et al Genetic basis of thoracic aortic aneurysms and dissections: focus on smooth muscle cell contractile dysfunction. Annu Rev Genomics Hum Genet. 2008;9:283–302. Epub 2008/06/12. doi: 10.1146/annurev.genom.8.080706.092303 . 18544034 10.1146/annurev.genom.8.080706.092303

[7] Humphrey JD, Schwartz MA, Tellides G, Milewicz DM. Role of mechanotransduction in vascular biology: focus on thoracic aortic aneurysms and dissections. Circ Res. 2015;116(8):1448–61. Epub 2015/04/11. doi: 10.1161/CIRCRESAHA.114.304936 ; PubMed Central PMCID: PMCPMC4420625. 25858068 10.1161/CIRCRESAHA.114.304936 PMC4420625

[8] Qiu H, Zhu Y, Sun Z, Trzeciakowski JP, Gansner M, Depre C, et al Short Communication: Vascular Smooth Muscle Cell Stiffness As a Mechanism for Increased Aortic Stiffness With Aging. Circ Res. 2010;107(5):615–9. doi: 10.1161/CIRCRESAHA.110.221846 20634486 10.1161/CIRCRESAHA.110.221846 PMC2936100

[9] Gao YZ, Saphirstein RJ, Yamin R, Suki B, Morgan KG. Aging impairs smooth muscle-mediated regulation of aortic stiffness: a defect in shock absorption function? Am J Physiol Heart Circ Physiol. 2014;307(8):H1252–61. Epub 2014/08/17. doi: 10.1152/ajpheart.00392.2014 ; PubMed Central PMCID: PMCPMC4200340. 25128168 10.1152/ajpheart.00392.2014 PMC4200340

[10] Sehgel NL, Vatner SF, Meininger GA. "Smooth Muscle Cell Stiffness Syndrome"-Revisiting the Structural Basis of Arterial Stiffness. Front Physiol. 2015;6:335 Epub 2015/12/05. doi: 10.3389/fphys.2015.00335 ; PubMed Central PMCID: PMCPMC4649054. 26635621 10.3389/fphys.2015.00335 PMC4649054

[11] Hartshorne DA. Biochemistry of the contractile process in smooth muscle Physiology of the Gastrointestinal Tract. Second Edition ed. New York: Raven Press; 1987 p. 423–82.

[12] Somlyo AP, Somlyo AV. Signal transduction by G-proteins, rho-kinase and protein phosphatase to smooth muscle and non-muscle myosin II. J Physiol. 2000;522(Pt 2):177–85. Epub 2000/01/19. doi: 10.1111/j.1469-7793.2000.t01-2-00177.x ; PubMed Central PMCID: PMCPMC2269761. 10639096 10.1111/j.1469-7793.2000.t01-2-00177.x PMC2269761

[13] Kitazawa T, Kitazawa K. Size-dependent heterogeneity of contractile Ca^2+^ sensitization in rat arterial smooth muscle. J Physiol. 2012;590(Pt 21):5401–23. Epub 2012/08/30. doi: 10.1113/jphysiol.2012.241315 ; PubMed Central PMCID: PMCPMC3515827. 22930267 10.1113/jphysiol.2012.241315 PMC3515827

[14] Kuang SQ, Kwartler CS, Byanova KL, Pham J, Gong L, Prakash SK, et al Rare, nonsynonymous variant in the smooth muscle-specific isoform of myosin heavy chain, MYH11, R247C, alters force generation in the aorta and phenotype of smooth muscle cells. Circ Res. 2012;110(11):1411–22. Epub 2012/04/19. doi: 10.1161/CIRCRESAHA.111.261743 ; PubMed Central PMCID: PMC22511748 22511748 10.1161/CIRCRESAHA.111.261743 PMC3917690

[15] Gao N, Huang J, He W, Zhu M, Kamm KE, Stull JT. Signaling through myosin light chain kinase in smooth muscles. J Biol Chem. 2013;288(11):7596–605. Epub 2013/01/31. doi: 10.1074/jbc.M112.427112 ; PubMed Central PMCID: PMCPMC3597801. 23362260 10.1074/jbc.M112.427112 PMC3597801

[16] Kamm KE, Stull JT. The function of myosin and myosin light chain kinase phosphorylation in smooth muscle. Annu Rev Pharmacol Toxicol. 1985;25:593–620. doi: 10.1146/annurev.pa.25.040185.003113 2988424 10.1146/annurev.pa.25.040185.003113

[17] Somlyo AP, and Somlyo A.V. Signal transduction and regulation in smooth muscle. Nature. 1994;372:231–6. doi: 10.1038/372231a0 7969467 10.1038/372231a0

[18] He WQ, Peng YJ, Zhang WC, Lv N, Tang J, Chen C, et al Myosin light chain kinase is central to smooth muscle contraction and required for gastrointestinal motility in mice. Gastroenterology. 2008;135(2):610–20. Epub 2008/07/01. doi: 10.1053/j.gastro.2008.05.032 ; PubMed Central PMCID: PMC2648853. 18586037 10.1053/j.gastro.2008.05.032 PMC2648853

[19] Zhang WC, Peng YJ, Zhang GS, He WQ, Qiao YN, Dong YY, et al Myosin light chain kinase is necessary for tonic airway smooth muscle contraction. J Biol Chem. 2010;285(8):5522–31. Epub 2009/12/19. doi: 10.1074/jbc.M109.062836 ; PubMed Central PMCID: PMC2820780. 20018858 10.1074/jbc.M109.062836 PMC2820780

[20] He WQ, Qiao YN, Zhang CH, Peng YJ, Chen C, Wang P, et al Role of myosin light chain kinase in regulation of basal blood pressure and maintenance of salt-induced hypertension. Am J Physiol Heart Circ Physiol. 2011;301(2):H584–91. Epub 2011/05/17. doi: 10.1152/ajpheart.01212.2010 . 21572007 10.1152/ajpheart.01212.2010 PMC3154661

[21] Kamm KE, Stull JT. Dedicated myosin light chain kinases with diverse cellular functions. J Biol Chem. 2001;276(7):4527–30. doi: 10.1074/jbc.R000028200 11096123 10.1074/jbc.R000028200

[22] Somlyo AP, Somlyo AV. Ca^2+^-sensitivity of smooth and non-muscle myosin II: modulation by G Proteins, kinases and myosin phosphatase. Physiol Rev. 2003;83:1325–58. doi: 10.1152/physrev.00023.2003 14506307 10.1152/physrev.00023.2003

[23] Isotani E, Zhi G, Lau KS, Huang J, Mizuno Y, Persechini A, et al Real-time evaluation of myosin light chain kinase activation in smooth muscle tissues from a transgenic calmodulin-biosensor mouse. Proc Natl Acad Sci USA. 2004;101:6279–84. doi: 10.1073/pnas.0308742101 15071183 10.1073/pnas.0308742101 PMC395960

[24] Ding HL, Ryder JW, Stull JT, Kamm KE. Signaling processes for initiating smooth muscle contraction upon neural stimulation. J Biol Chem. 2009;284(23):15541–8. Epub 2009/04/08. doi: 10.1074/jbc.M900888200 ; PubMed Central PMCID: PMC2708850. 19349274 10.1074/jbc.M900888200 PMC2708850

[25] Tsai MH, Kamm KE, Stull JT. Signalling to contractile proteins by muscarinic and purinergic pathways in neurally stimulated bladder smooth muscle. J Physiol. 2012;590(Pt 20):5107–21. Epub 2012/08/15. doi: 10.1113/jphysiol.2012.235424 ; PubMed Central PMCID: PMC3497566. 22890701 10.1113/jphysiol.2012.235424 PMC3497566

[26] Morano I, Chai GX, Baltas LG, Lamounier-Zepter V, Lutsch G, Kott M, et al Smooth-muscle contraction without smooth-muscle myosin. Nat Cell Biol. 2000;2(6):371–5. doi: 10.1038/35014065 . 10854329 10.1038/35014065

[27] Lofgren M, Ekblad E, Morano I, Arner A. Nonmuscle myosin motor of smooth muscle. J Gen Physiol. 2003;121(4):301–10. doi: 10.1085/jgp.200208720 . 12668734 10.1085/jgp.200208720 PMC2217371

[28] Yuen SL, Ogut O, Brozovich FV. Nonmuscle myosin is regulated during smooth muscle contraction. Am J Physiol Heart Circ Physiol. 2009;297(1):H191–9. doi: 10.1152/ajpheart.00132.2009 19429828 10.1152/ajpheart.00132.2009 PMC2711724

[29] Brozovich FV, Nicholson CJ, Degen CV, Gao YZ, Aggarwal M, Morgan KG. Mechanisms of Vascular Smooth Muscle Contraction and the Basis for Pharmacologic Treatment of Smooth Muscle Disorders. Pharmacol Rev. 2016;68(2):476–532. Epub 2016/04/03. doi: 10.1124/pr.115.010652 ; PubMed Central PMCID: PMCPMC4819215. 27037223 10.1124/pr.115.010652 PMC4819215

[30] Wendling O, Bornert JM, Chambon P, Metzger D. Efficient temporally-controlled targeted mutagenesis in smooth muscle cells of the adult mouse. Genesis. 2009;47(1):14–8. Epub 2008/10/23. doi: 10.1002/dvg.20448 . 18942088 10.1002/dvg.20448

[31] Wirth A, Benyo Z, Lukasova M, Leutgeb B, Wettschureck N, Gorbey S, et al G12-G13-LARG-mediated signaling in vascular smooth muscle is required for salt-induced hypertension. Nat Med. 2008;14(1):64–8. Epub 2007/12/18. doi: 10.1038/nm1666 . 18084302 10.1038/nm1666

[32] He WQ, Qiao YN, Peng YJ, Zha JM, Zhang CH, Chen C, et al Altered contractile phenotypes of intestinal smooth muscle in mice deficient in myosin phosphatase target subunit 1. Gastroenterology. 2013;144(7):1456–65, 65 e1-5. Epub 2013/03/19. doi: 10.1053/j.gastro.2013.02.045 ; PubMed Central PMCID: PMCPMC3782749. 23499953 10.1053/j.gastro.2013.02.045 PMC3782749

[33] Tsai MH, Chang AN, Huang J, He W, Sweeney HL, Zhu M, et al Constitutive phosphorylation of myosin phosphatase targeting subunit-1 in smooth muscle. J Physiol. 2014;592(Pt 14):3031–51. Epub 2014/05/20. doi: 10.1113/jphysiol.2014.273011 ; PubMed Central PMCID: PMC4214658. 24835173 10.1113/jphysiol.2014.273011 PMC4214658

[34] Gao N, Chang AN, He W, Chen CP, Qiao YN, Zhu M, et al Physiological signalling to myosin phosphatase targeting subunit-1 phosphorylation in ileal smooth muscle. J Physiol. 2016;594(12):3209–25. Epub 2016/02/06. doi: 10.1113/JP271703 ; PubMed Central PMCID: PMCPMC4908025. 26847850 10.1113/JP271703 PMC4908025

[35] Kamm KE, Hsu LC, Kubota Y, Stull JT. Phosphorylation of smooth muscle myosin heavy and light chains. Effects of phorbol dibutyrate and agonists. J Biol Chem. 1989;264(35):21223–9. 2592371

[36] Persechini A, Kamm KE, Stull JT. Different phosphorylated forms of myosin in contracting tracheal smooth muscle. J Biol Chem. 1986;261(14):6293–9. 3516992

[37] Mizuno Y, Isotani E, Huang J, Ding H, Stull JT, Kamm KE. Myosin light chain kinase activation and calcium sensitization in smooth muscle *in vivo*. Am J Physiol Cell Physiol. 2008;295:C358–C64. PubMed Central PMCID: PMCPMC2518426. doi: 10.1152/ajpcell.90645.2007 18524939 10.1152/ajpcell.90645.2007 PMC2518426

[38] Gunst SJ, Zhang W. Actin cytoskeletal dynamics in smooth muscle: a new paradigm for the regulation of smooth muscle contraction. Am J Physiol Cell Physiol. 2008;295(3):C576–87. Epub 2008/07/04. 00253.2008 [pii] doi: 10.1152/ajpcell.00253.2008 ; PubMed Central PMCID: PMC2544441. 18596210 10.1152/ajpcell.00253.2008 PMC2544441

[39] Gabella G. Structural apparatus for force transmission in smooth muscles. Physiol Rev. 1984;64(2):455–77. Epub 1984/04/01. doi: 10.1152/physrev.1984.64.2.455 . 6369351 10.1152/physrev.1984.64.2.455

[40] Karimi A, Milewicz DM. Structure of the Elastin-Contractile Units in the Thoracic Aorta and How Genes That Cause Thoracic Aortic Aneurysms and Dissections Disrupt This Structure. Can J Cardiol. 2016;32(1):26–34. Epub 2016/01/03. doi: 10.1016/j.cjca.2015.11.004 ; PubMed Central PMCID: PMCPMC4839280. 26724508 10.1016/j.cjca.2015.11.004 PMC4839280

